# Supplementary material for: Beyond Neural Noise: Critical Dynamics Predict Slower Reaction Times in Adults With and Without ADHD
Source: bioRxiv. 2026 Mar 17:2026.03.13.711705. Preprint. [Version 1] doi: 10.64898/2026.03.13.711705 (PMC13015431; doi:10.64898/2026.03.13.711705)
Supplement: Supplement 1 [file media-1.pdf]

Appendix A. FOOOF Results

The spectral slope was also determined using spectral parameterization or the specparam (formerly known as "Fitting Oscillations and One-Over-f" or FOOOF) toolbox [64]. The power spectral density was first calculated for each trial using the multitaper method and then the slope was fit with specparam using the following parameter settings: peak_width_limits = [2,7], max_n_peaks = 5, min_peak_height = 0.05, peak_threshold = 0.2, aperiodic_mode = 'fixed'.

During the auditory task, the spectral slopes showed a pattern consistent with the LRTCs, with a main effect of event type ($F(1.97, 229.27) = 7.60, p = 0.001$). None of the post hoc comparisons were significant, but the pairwise comparisons between fast vs slow and passive viewing, and average vs passive viewing were trend-level ($p < 0.1$). During the auditory task, spectral slopes also showed a main effect of diagnosis ($F(1, 135) = 4.943, p = 0.028$) where individuals with ADHD had steeper slopes than the control group overall. During the visual task, spectral slopes also showed a significant main effect of diagnosis during the visual task in the occipital electrodes ($F(1, 131) = 4.79, p = 0.043$), indicating once again that ADHD was associated with steeper slopes than control group overall (Fig A.7).

The differences between the FOOOF and Wavelet based slopes is likely due to signal-to-noise (SNR) and, for FOOOF, model fit. Slopes are calculated on every trial instead of the mean PSD of multiple trials which decrease the SNR which in turn affects the model fit for the FOOOF algorithm as it tries to distinguish between periodic and aperiodic behavior. Meanwhile, the discrete wavelet method (DWT) used includes a de-noising step that may help diminish the number of sharp transients and emphasize the broadband scaling trend. The choice between the use of either DWT or FOOOF for calculation of the spectral slope ultimately comes down to whether there is an interest in looking at both the slope and oscillations and the SNR of the underlying signal.

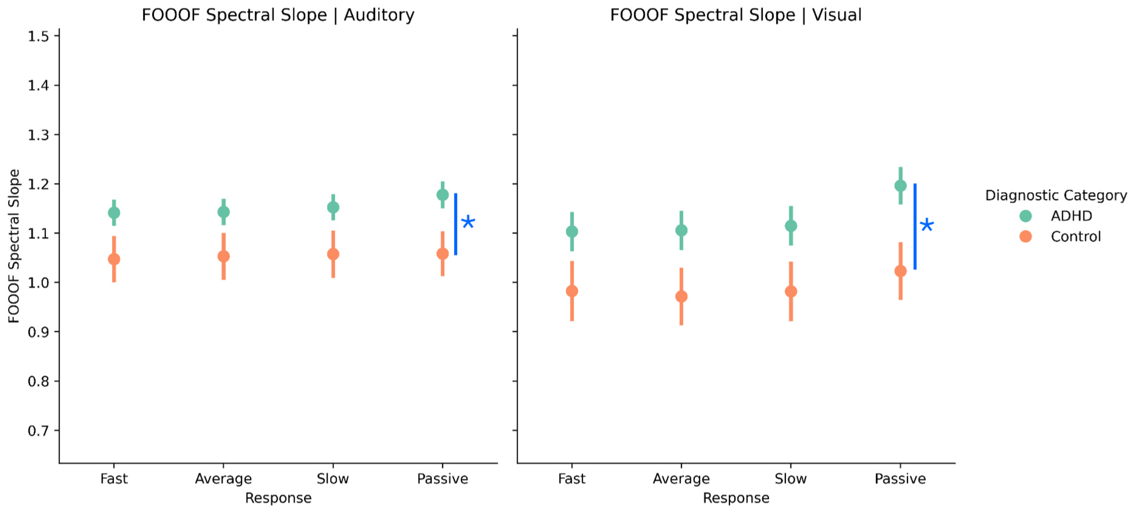

Figure A.7: **FOOOF Slopes are steeper in ADHD at rest.** *Note:* The colors of the bars indicate whether the effect is due to an interaction (orange) or a main effect of response (purple) or diagnosis (blue). The asterisks above the bars denote significance (* $p < 0.05$, ** $p < 0.001$, and *** $p < 0.001$). Error bars represent the standard error.

Appendix B. Criticality-associated EEG features were not associated with individual differences.

Given the relationship between the calculated EEG features and RT slowing was mirrored by the differences between ADHD and the control group (even though they were in the opposite direction as expected), we lastly looked to see if this pattern of results extended to other individual differences in behavioral performance and ADHD symptoms. We also wanted to compare the results of these correlations with those between a well-known metric in the ADHD literature that has previously been used as a diagnostic aid: the theta/beta ratio (TBR). To do so, we ran Spearman's rank correlation analyses between the z-scored EEG metrics for each response type (fast, average, slow and passive), five performance metrics (mean RT, median RT, RT standard deviation, hit rate, and percent correct), and the ASRS hyperactive and

inattentive subscale scores. While spectral slope and LRTCs did not show any significant correlations, the oscillatory metrics (low-frequency variability and power and TBR) did appear to correlate with hyperactivity symptoms and behavioral accuracy. However, none of these correlations survived a subsequent correction for family-wise error.
